# Supplementary material for: Multicoding in neural information transfer suggested by mathematical analysis of the frequency-dependent synaptic plasticity in vivo
Source: Sci Rep. 2020 Aug 18;10:13974. doi: 10.1038/s41598-020-70876-4 (PMC7435278; doi:10.1038/s41598-020-70876-4)
Supplement: Supplementary file 1 — Supplementary information. [file 41598_2020_70876_MOESM1_ESM.pdf]

Supplementary Information for: Multicoding in neural information transfer suggested by mathematical analysis of the frequency-dependent synaptic plasticity in vivo

Katsuhiko Hata, Osamu Araki, Osamu Yokoi, Tatsumi Kusakabe, YoshioYamamoto, Susumu Ito, and Tetsuro Nikuni

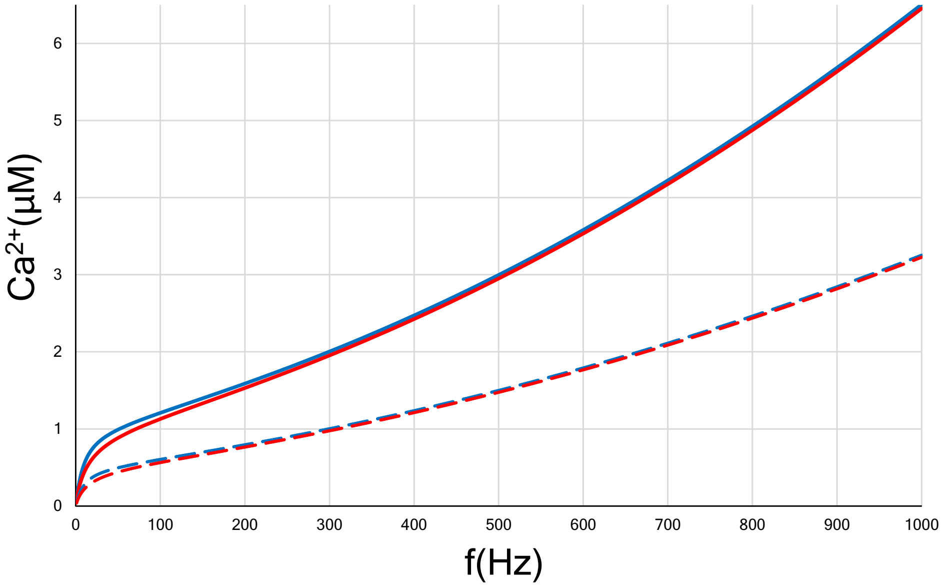

Figure S 1: Frequency dependence of the postsynaptic calcium concentration from 0 to 1000 Hz. In each graph, blue solid, red solid, blue dotted, red dotted lines indicate constant-ISI input to τ_{Ca} = 80 ms neurons, Poisson input to τ_{Ca} = 80 ms neurons, constant-ISI input to τ_{Ca} = 40 ms neurons and Poisson input to τ_{Ca} = 40 ms neurons, respectively.

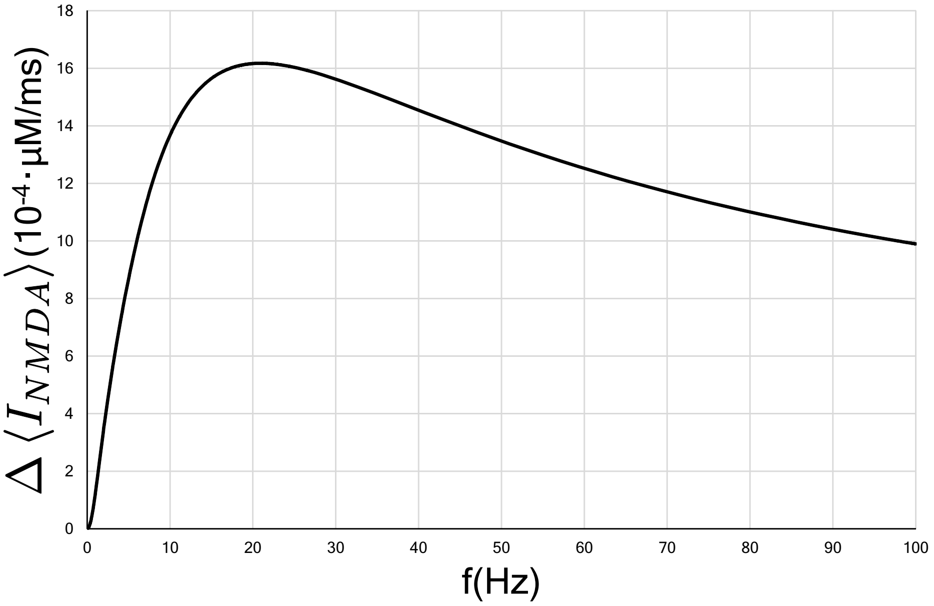

Figure S 2: A plot of $\Delta \langle I_{NMDA}(f) \rangle$ which is defined by Eq. (8). The x axis indicates the input frequency, and the y axis represents the statistical average of the difference in I_{NMDA} at the post-synapse between constant-ISI and Poisson stimulation.
